# Tumor Vessels Fuel the Fire in Glioblastoma

**DOI:** 10.3390/ijms22126514

**Published:** 2021-06-17

**Authors:** Sara Rosińska, Julie Gavard

**Affiliations:** 1 CRCINA, Inserm, CNRS, Université de Nantes, 44000 Nantes, France; Sara.Rosinska@univ-nantes.fr; 2 Integrated Center for Oncology, ICO, 44800 St. Herblain, France

**Keywords:** neovascularization, angiogenesis, co-option, vasculogenesis, endothelial cells, vascular mimicry, cancer, glioblastoma, vessel, cancer stem cells

## Abstract

Glioblastoma, a subset of aggressive brain tumors, deploy several means to increase blood vessel supply dedicated to the tumor mass. This includes typical program borrowed from embryonic development, such as vasculogenesis and sprouting angiogenesis, as well as unconventional processes, including co-option, vascular mimicry, and transdifferentiation, in which tumor cells are pro-actively engaged. However, these neo-generated vascular networks are morphologically and functionally abnormal, suggesting that the vascularization processes are rather inefficient in the tumor ecosystem. In this review, we reiterate the specificities of each neovascularization modality in glioblastoma, and, how they can be hampered mechanistically in the perspective of anti-cancer therapies.

## 1. Introduction

Glioblastoma (GBM) is the most aggressive type of brain cancers in adults with high recurrence and mortality rate. The standard-of-care therapy for GBM encompasses multimodal approaches, including surgery, radiotherapy, and chemotherapy (with the alkylating agent temolozomide, TMZ) [1,2]. Like many other solid tumors, GBM expansion relies on a dedicated blood supply, which seeds from the cerebral vasculature, a dense and specialized network. A better understanding of how tumor cells hijack and recompose the brain endothelium to their own benefit is crucial to the design of more effective therapies.

The infiltrative and resistant nature of GBM might come from a subpopulation of tumor cells with stem-like properties, the so-called glioblastoma stem-like cells (GSCs). GSCs exhibit pluripotent characteristics with self-renewal ability, multipotency, and chemo/radio-resistance. GSCs are reported to recapitulate tumor initiation, invasion, and relapse [3,4,5,6,7]. Alongside cancer progression and invasion, GSCs can differentiate towards different cell types and evolve into various, intermediate stages to adapt to their niche. Depending of their microenvironment and their own intrinsic properties, some differentiated GSCs remain able to maintain their stemness properties and consequently re-initiate tumor after surgery [8]. Of note, GSCs reside in specific habitats within the tumor mass: perivascular niche, hypoxic/necrotic core, and invasive front. The interactions between GSCs and their niche components hinge on cellular communication through cell-cell contacts, stroma, and soluble factors, which in turn determine GSC fate and contribute to tumor heterogeneity [8]. First, in the perivascular niche, essentially formed by endothelial cells (ECs), GSCs are mostly found near vessels [7,9]. This privileged interaction assists the stemness properties, self-renewal, and proliferation of GSCs [10,11]. Conversely, studies revealed that GSCs can be involved in the formation of new blood vessels [12]. Second, the hypoxic/necrotic core promotes the formation of new blood vessels, through multiple processes, including angiogenesis, vasculogenesis, and transdifferentiation (please see details below). Moreover, this hypoxic/necrotic niche orchestrates GSC proliferation and their resistance to therapies. Not only does hypoxic-based nutrient restriction provoke adaptive mechanisms in GSCs towards aerobic glycolysis [13], but this also results in their quiescence, a state that further contributes to chemo- and radio-resistance [14,15]. Under hypoxic conditions, quiescent GSCs might also be rerouted towards more mesenchymal-like features, causing enhanced migration and invasion [16,17]. The third habitat in which GSCs can locate is the invasive front, localized on the edge of the tumor bulk. Pre-existing vessels from the healthy parenchyma serve as tracks for GSC invasion, further exacerbating a spreading and infiltrative phenotype [18] in a phenomenon coined under the term co-option (please see details below).

The present review will cover the diversity of neovascularization processes in glioma, including co-option, angiogenesis, vasculogenesis, vascular mimicry, and the recently described transdifferentiation phenomenon. Interaction with the glymphatic system will not be detailed here. A general view on the molecular mechanisms relying on GSCs and their non-GSC counterparts (named here as GBM cell population) will be provided, together with future perspectives in therapies.

## 2. Main Features of Tumor Vessels in Glioblastoma

**Figure 1:**
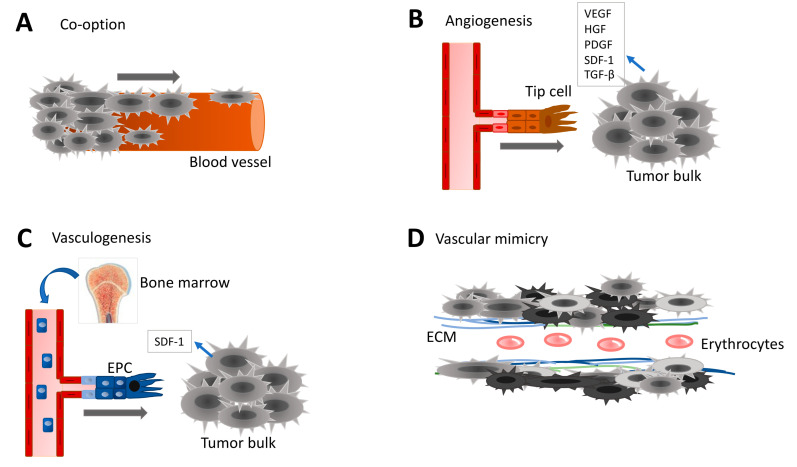
Different types of neovascularization occurring in GBM. (**A**) Vascular co-option of tumor cells migrating on existing blood vessels contributes to tumor spreading and invasion. Co-option requires direct binding between cancer cells and either endothelial cells (ECs), pericytes or extracellular matrix (ECM). (**B**) Angiogenesis corresponds to the creation of new blood vessels from pre-existing vascular network in order to increase oxygen and nutrient supply to the growing tumor mass. The ‘tip cell’ is attracted towards the tumor mass and takes the lead in the sprouting newly emerging vessel. Angiogenic factors include Vascular Endothelial Growth Factor (VEGF), Hepatocyte-derived Growth Factor (HGF), Platelet-Derived Growth Factor (PDGF), Stromal Derived Factor (SDF-1), and Transforming Growth Factor (TGF). (**C**) Endothelial progenitor cells (EPCs) originated from the bone marrow are circulating in the blood stream. Cancer cells recruit EPCs to participate in neovascularization. Often, the vasculogenesis is followed with angiogenesis. (**D**) Vascular mimicry aims at improving blood irrigation into the growing tumor mass. Cancer cells or cancer stem-like cells contribute to the formation of functional tubular-like structures. Capacity of cancer stem-like cells to transdifferentiate towards pericyte-like or EC-like is one of the key elements of vascular mimicry.

The cerebral vasculature is composed of well-organized, multicellular functional units that ensure efficient irrigation and drainage in the central nervous system. Quiescent ECs organize as a monolayer with a streamlined surface and linked via junctional molecules. ECs are further ensheathed by pericytes and surrounded by a specialized basal lamina, shared with pericytes and astrocytic end-feet, and sparsely interconnected with neuronal ending and microglia [19]. The tumor ecosystem dramatically affects this stereotyped vasculature, which ultimately features tortuous, disorganized vessels, lacking physiological regulation and controlled architecture [20]. These tumor vessels also exhibit zones with larger diameters and denser basement membranes, while both the endothelial wall and pericyte coverage are abnormal [21]. As a consequence, the blood-brain barrier (BBB) is not preserved and tumor vessels are highly permeable and leaky [22,23]. Increased permeability is associated with higher edema, inflammation, and enhanced immune cell infiltration. Despite the elevated number of ECs in the tumor mass, hypoxia remains. Because the abnormal vasculature system cannot be fully functional, the delivery of chemotherapeutic agents to the tumor mass is also limited [24]. The whole tumor microenvironment constantly adapts and educates through several reciprocal mechanisms in order to build new vessels (i.e., neovascularization). The isocitrate dehydrogenase 1 (IDH1) mutation is one of the most critical genomic alterations that can discriminate between lower grade and malignant glioma. Thus, IDH1 status (either mutant or wild-type, WT) is associated with differential pericyte coverage, perfusion and vascular gene expression signature [25,26,27]. Likewise, whether a common genomic signature and/or molecular subtypes may also impact on the formation and functionality of tumor vessels requires further investigation. Neovascularization in glioma covers multifaceted processes, including co-option, angiogenesis, vasculogenesis, vascular mimicry, and tumor cell transdifferentiation (Figure 1). This diversity impacts the way we think about anti-angiogenic therapies in cancer.

## 3. Vessel Co-Option

### 3.1. Definition and Concepts

Vessel co-option, also known as “angiotropism” or “perivascular/extravascular migration”, is a non-angiogenic process through which tumor cells utilize pre-existing blood vessels to support tumor growth, survival, and metastasis. Additionally, GBM cells, as well GSCs, can also employ white matter tracts as alternate spreading paths [28]. Because the brain is a highly vascularized organ, it offers favorable conditions for tumor cells to grow and expand in perivascular niches. Cancer cells can migrate along the abluminal surface of vessels and/or infiltrate the stromal space between vessels, ultimately leading to the incorporation of pre-existing vessels into the tumor [29]. Co-option is employed by tumor cells in two scenarios, either by the primary tumor cells to spread into the host tissue or by metastatic cells following their extravasation from the vascular tubes in order to colonize new tissues [30]. Interestingly, this process appears to be vascular endothelial growth factor (VEGF)-independent and occurs in the absence of typical angiogenic processes [31]. In these conditions, VEGF inhibitors fail to dampen tumor cell progression and invasion, as reported in orthotopic mouse models for GBM [31].

Tissue infiltration occurs either via single cancer cell co-option along blood vessel or collectively as perivascular groups of cells on vascular tracks [32]. Multiscale analysis, notably using intravital imaging, revealed direct interactions between vascular cells and tumor cells, suggesting that the latter exploit blood vessels and invade the brain tissue following such paths [33,34]. For instance, by monitoring glioma cell invasion in real-time using multiphoton laser scanning microscopy in a syngeneic model (i.e., mouse GBM cell line GL261 intracranial injection), Winkler et al. documented the dynamics of host brain vessel co-option and established that glioma cell invasion was faster and more efficient along brain microvessels [33]. Of note, C6 rat glioma cells co-opted brain vessels at early stages soon after their orthotopic injection [35]. Likewise, orthotopically xenografted GBM cells spread quickly following their injection in an angiogenesis-independent manner. In keeping with this idea, serial transplantation of human-derived GBM cells highlighted that early passaged tumor cells co-opted the brain vasculature, while at later passages, angiogenic processes were preferred [36].

GBM cells are able to associate with blood vessels from all calibers and types, may displace astrocyte end-feet from the vasculature and are in direct contact with ECs. Ultimately, this behavior results in loss of astrocyte-vascular coupling, BBB breakdown and increased vessel leakage [34]. In vivo studies unveiled that GBM cells tethered to pericytes by forming actin-rich protrusions and modified their contractility, in a cdc42 and CD44-dependent manner [37]. However, this undesired contact with tumor cells alters further on pericyte functions and ultimately affects the BBB integrity. In vivo studies combined with mathematical modeling revealed that co-opting tumor cells compressed host vessels, reducing flow and thereby causing local hypoxia [38].

### 3.2. Mechanistic Insights in Vessel Co-Option

Direct physical contacts between cancer cells and the vascular tracks, including ECs, pericytes, and extracellular matrix (ECM), are required in the course of co-option [35]. These cell-to-cell and cell-to-ECM interactions are crucial to deploy co-option phenotype of tumor cells. Adhesion molecules, including different integrins and L1CAM, and receptors like CXC chemokine receptors (CXCR) and Epithelial Growth Factor Receptor (EGFR) are employed [36,39]. Accordingly, GBM cells expressing β1-integrin bind to different matrix proteins composing the basal lamina of brain capillaries, among which are collagens I and IV, fibronectin, laminin, and vitronectin [30,37]. GBM cells localized in perivascular niches express high levels of αvβ8-integrin, which promotes tumor cell anchoring to blood vessels, co-option, and tumor progression. Alternatively, GSCs expressing L1CAM bind directly to αvβ3 integrin expressed at the surface of ECs [40]. Moreover, metastatic cancer cells found in the brain also employ similar adhesion molecules, namely L1CAM and β1-integrin, in order to both tether to brain capillaries and spread along microvessels [41,42,43].

Additional membrane receptors from either the endothelial or tumor sides are at play in co-option. Xenograft and syngeneic models suggested that the Stromal Derived Factor (SDF-1) receptor CXCR4 is up-regulated in GBM samples, while its knockdown inhibited both the growth and the perivascular invasion of tumor cells [44]. Likewise, the level of expression of the constitutively active mutant vIII of EGFR (EGFRvIII) determines the fate of GBM progression and co-option, as a high level of this variant is associated with strong cell proliferation and collective cell migration. Conversely, a low level of EGFRvIII promotes single cell migration associated with an enhanced infiltrative phenotype [45]. Additional studies suggest that ECs themselves might engage GSCs in invasion along vessel tracks by secreting attractive molecules, such as interleukin 8 (IL-8) [46]. Likewise, EC-produced cytokines provoke chemotaxis and the co-option of GBM cells expressing the B2R bradykinin receptor [47,48].

In order to move along vessels, co-opting tumor cells undergo morphological and molecular changes. GBM cells expand their invasive and migratory abilities, together with the emergence of a mesenchymal-like phenotype, which encompasses cytoskeleton rearrangement, cell adhesion remodeling, and signaling dysregulations. This mimicking process of epithelial to mesenchymal transition (EMT) can be termed as “EMT-like” or “glial-to-mesenchymal transition” (GMT) [49,50]. It is not entirely clear whether this EMT-like/GMT-driven co-option is an exclusive ability of GSCs or whether more differentiated GBM cells can also undergo similar transitions. The role of EMT-like/GMT in vessel co-option is poorly understood and not fully confirmed, despite shared mechanisms between them. For example, hypoxia induces the up-regulation of the typical EMT transcription factor ZEB2 (zinc finger e-box binding homeobox 2), which leads to a decreased level of EphrinB2 and enhanced invasion in GBM cells [51]. Of note, EphrinB2 has been reported to drive perivascular invasion [52]. In organotypic brain slice culture, tumor cells form membrane extensions via cdc42- and actin-dependent mechanisms that allow blood vessel co-option and modify pericytes [37].

Furthermore, it has been suggested that the molecular signature of glioma cells determines the type of glioma-vasculature interactions [53]. Indeed, Olig2^+^ oligodendrocyte precursor-like glioma cells invade the parenchyma by single-cell vessel co-option and do not affect the underlying vasculature. Conversely, Olig2^−^ glioma cells grow as perivascular clusters, leading to disruption of BBB and activation of an innate immunity response. While co-opting tumor cells escape the anti-VEGF and TMZ therapies, systemic Wnt7 inhibition reduces vessel co-option of tumor cells and potentiates TMZ effect [53]. Other pathways might dictate the invasive nature. For instance, GSCs exhibit more perivascular invasive phenotype when the RNAse activity of the endoplasmic reticulum-resident transmembrane protein IRE1α (inositol-requiring transmembrane kinase/endoribonuclease 1α) is abrogated [54].

Alternate mechanism involved in co-option might rely on the formation of interconnected intercellular network using ultra-long actin-rich tumor microtubes (TMs) [55]. In this context, glioma-emanating TMs engage cellular interactions between cancer cells together, and, between cancer cells and the brain vasculature [55]. Recently, it has been shown in vivo that NOTCH1 is an important modulator of TM-network formation in glioma cells [56]. The downregulation of the NOTCH1 pathway exhaust the pool of glioma cells associated with perivascular microenvironment and diminishes the niche protective action. However, NOTCH1 depletion induces TM-formation and reinforces resistance to therapy. The discovery of TM-network of glioma in interaction with the perivascular niche provides novel concepts to target GBM [56]. Several experimental data also support the notion that tumor cell co-option in perivascular niches contributes in tumor resistance and recurrence. First, this system allows to create tumor extensions beyond the surgical margin. Moreover, co-option might emerge as a strategy deployed by tumor cells in response to anti-angiogenic therapies [57]. Because co-option is not an angiogenic process per se, tumor cells might evade anti-VEGF therapies using existing vasculature. Subsequently, the fraction of co-opted vessels augments upon anti-VEGF therapeutic challenge in several tumor models, including GBM xenografts [40,58].

## 4. Sprouting Angiogenesis

### 4.1. Pathological Angiogenesis

Angiogenesis is a multi-step, highly controlled and complex process occurring in physiological conditions (i.e., during development, perinatal development and in adults), as well as in pathological conditions, especially in cancer. Angiogenesis can be defined as the formation of new blood vessels from pre-existing ones, and involves proliferation, migration, and differentiation of vascular ECs. Angiogenesis is a tightly tuned process regulated by a balance between pro-angiogenic and inhibiting factors (Table 1) [59,60,61,62,63,64,65,66,67,68,69,70,71,72]. Among the broad range of angiogenic stimuli, a lack of oxygen is one of the more potent triggers, which mainly operates via the activation of the hypoxia-inducible factor (HIF-1) transcription factor and commands the expression of VEGF [59].

Upon physiological angiogenic stimulation, ECs and pericytes dynamically remodel, with striking changes in their cellular interactions, based on active proteolytic degradation of the basement membrane and weakening of endothelial junctions (VE-cadherin, occludin). ECs might ultimately specialize in either tip or stalk cells, involving a finely-tuned choreography of DLL-4 (Delta-like 4)/NOTCH and VEGF/VEFGR2 signaling [73]. Active tip cells sense their stromal environment by extending filipodia and further guide endothelial tube-like structures, under the impulsion of high rate of proliferation in the stalk cells at the rear. Tip and stalk cells continuously compete for the tip cell position controlled by differential VEGFR expression level in individual ECs [74,75]. Next, secretion of platelet-derived growth factor (PDGF) and activation of angiopoietin (Ang) receptor Tie-2, allows the recruitment of pericytes and mural cells (smooth muscle cells), later producing new ECM [67]. In the tumor ecosystem, this angiogenesis mechanism is handicapped as tumor cells, and other cellular components of the tumor mass, secrete abnormal, uncontrolled, level of proangiogenic factors [24]. Using similar sprouting angiogenic mechanisms, there are outrageous numbers of ECs that serve as tip cells, leading to a disorganized pattern of neovessels [60]. Among the external cues involved, VEGF is highly up-regulated in cancer cells. Hence, VEGF becomes the central target for the development of anti-angiogenic therapy in GBM [76].

### 4.2. Mechanisms of Cell-Cell Communication during Angiogenesis

The intercellular communications between tumor cells and their vascular microenvironment and associated cellular components (ECs, pericytes, fibroblasts, macrophages) dictate angiogenesis. This occurs via several means, such as secreted soluble factors, extracellular vesicles (EVs), and actin-rich protrusions.

Most of the secreted soluble factors operate as cytokines on their cognate receptors. This includes VEGF (VEGF-A, -B, -C), PDGF, SDF-1, and the transforming growth factor (TGF-β). Moreover, tumor cells can promote angiogenesis via the secretion of matrix metalloproteases MMPs (notably MMP3, MMP7), which remodel the ECM, and subsequently alter its composition and stiffness [77].

Alternatively, EVs have emerged as potent communication tools that may act locally in a paracrine way, and at distance throughout the organism. These small membrane vesicles are heterogeneous in origin, size, and quality. GBM-derived EVs are detectable in plasma and cerebrospinal fluid of GBM patients and contain a wide variety of biological materials (proteins, miRNA, mRNA, DNA, lipids, and metabolites) [78]. It has been shown that GBM- and/or GSC-derived EVs transport oncogenic information as well as pro-angiogenic cues [79,80,81,82,83,84,85]. EVs from GBM-derived cultured cells, plasma, and cerebrospinal fluid exert pro-angiogenic action on brain ECs, in terms of proliferation, migration, sprouting, and tube formation [79,86,87]. Importantly, the plasmatic concentrations in nanoparticles (also referred as vesiclemia) are higher in GBM patients [78,80,88].

Recently, direct intercellular contacts have been described in GBM via actin-rich protrusions that are able to connect two separated cells, and were named as tunneling nanotubes (TNT) or TMs. TNTs are long-range intercellular cytoplasmic channels for direct cell-to-cell communication. Such structures allow the rapid exchange of cellular cargos between connected, non-adjacent cells, including organelles, vesicles, molecules, ions and pathogens [55,89]. It has been shown that glioma cells can create multicellular network-like TNT structures that contribute to increased resistance to radio/chemotherapy [55,90]. In vitro and in vivo studies report that pericyte-derived TNTs actively explore and sense their surrounding microenvironment, and connect to targeted vessels through pericyte-to-pericyte and/or pericyte-to-EC bounds. TNTs may have a primary role in the early phases of angiogenesis in the tumors from the central nervous system [91,92,93].

GSCs themselves express angiogenic markers, like VEGF and its receptors VEGFR1 and VEGFR2, as well as HIF-1α and HIF-2α under hypoxia conditions [94]. Soluble VEGF-derived from GSCs could trigger in vitro tubulogenesis of cultured ECs [4]. It is noteworthy that VEGF was also found in GSC-derived EVs [79]. Other studies also confirmed that GSCs secrete SDF-1, in addition to VEGF [95].

## 5. Vasculogenesis

Vasculogenesis emerges in GBM as an alternate mechanism for de novo blood vessel formation via the recruitment of circulating endothelial progenitor cells (EPC) (also known as bone marrow-derived cells, BMDC), whose circulating levels are elevated in cancer including GBM patients [96,97,98]. Although it seems that vasculogenesis has a modest contribution to tumor vascularization, the recruitment of EPCs is enhanced when angiogenesis is held in check due to anti-angiogenic treatment or radiotherapy [99]. In keeping with this idea, the inhibition of vasculogenesis, but not sprouting angiogenesis, prevents the recurrence of GBM after irradiation in mice [100].

Two independent mouse models showed that only advanced tumors recruit and incorporate bone marrow-derived EPCs into neovessels, through a multiple-step process regulated by a wide range of cytokines and chemokines secreted by tumor cells [101,102,103,104]. Moreover, the recruitment of EPCs was more pronounced in GSC-enriched tumors, as compared to non-GSC xenograft models [101]. In tumors, the hypoxia-dependent secretion of SDF-1 promotes the EPC recruitment to regions in the need for neovascularization [95,100,105]. EPCs occupy the hypoxic niche, where they rapidly adapt and produce reactive oxygen species (ROS) that up-regulate in turn proteases to degrade the surrounding ECM and facilitate the formation of EPC clusters [106]. As these clusters continue to degrade the neighborhood matrix, the number of incoming cells increases, and is stabilized through β2-integrin-, ICAM1- (intercellular adhesion molecule 1) and VE-cadherin-mediated adhesion. Last, recruited EPCs differentiate into mature ECs and engage with the surrounding stiffer ECM to sprout, secrete pro-angiogenic factors (IL-8 and CCL2), and initiate the formation of vascular networks [107].

## 6. Vascular Mimicry

### 6.1. Biomarkers of Vascular Mimicry

Originally described in invasive malignant melanoma, vascular mimicry (VM) is a process of formation of ‘vessel-like’ structures without ECs, characterized with matrix-rich layers and fluid-conducting tubes with a lumen, and further able to supply oxygen, nutrients and eliminate cell waste [108,109]. Although these microfluidic tubes remain elusive, they may be defined as follow: (i) absence of endothelial markers (CD34, CD31, von Willebrand factor vWF) on the inner wall, (ii) vascular-like channels are lined with tumor cells; (iii) positive for PAS (periodic acid–Schiff) staining, and (iv) presence of erythrocytes in their lumen [110,111]. However, VE-cadherin and EphA2 are expressed at higher levels in VM-positive glioma and were found required for VM network formation, especially under hypoxic conditions [112]. A meta-analysis of VM samples suggested that the most accurate method relies on CD31-/PAS+ rather than CD34-/PAS+ [113]. Moreover, VM might be of two kinds: a tubular type characterized by tumor cells lining the vessel-like structures, and a patterned matrix-type of secreting matrix proteins [111,114,115]. VM is thought to be employed in aggressive glioma, notably associated with hypoxia conditions [114,116,117]. Although more works are needed, VM does not necessarily correlate with the probability of survival [117,118,119,120].

### 6.2. Key Molecules and Signaling Pathways Involved in Vascular Mimicry

VM implies the progressive acquisition of EC–like functions, which hinges on the tumor microenvironment and tumor plasticity. Because VM formation compromises cellular information and alters morphology, proliferation, migration, matrix remodeling and adhesome [111,121], the cellular origin is under debate. For instance, tube-like structures might originate from transformed cancer cells or pluripotent cancer stem cells, while tumor-associated macrophages (TAMs) might be implicated [122,123,124].

Nonetheless, hypoxia, via its main effector HIF-1, appears as the most important factor in VM. HIF-1 directly regulates multiple VM-related molecules, such as VEGF, MMPs, TGF-β, and EMT transcription factors [83,125,126,127,128]. In GBM, TGFβ drives the expression of adhesion molecules, among which is VE-cadherin [129,130]. Indeed, VE-cadherin is highly expressed in VM-positive glioma and its expression correlates with glioma grade [131]. Furthermore, the VEGF/VEGFR2 pathway may be decisive for vascular-like channel formation that strongly relies on VEGFR2 [132,133]. Macrophage migration inhibition factor (MIF) was shown in vitro and in vivo to induce VM through the CXCR4/AKT signaling pathway [134]. Based on their role in matrix remodeling, MMPs are most likely involved in VM formation [121,135]. Other pathways, including the PI3K/mTOR pathway, insulin-like growth factor binding protein 2 (IGFBP2) and the transcription factor ETV2, have also been reported to promote the establishment of VM [136,137,138,139,140].

GSCs are at the upmost importance for VM formation, based notably on their high plasticity and differentiation ability into EC-like cells to form VM (please see below) [141].

## 7. Transdifferentiation of GSCs into Vascular-Like Cells

Similarly to neural stem cells (NSCs) that have the capacity to differentiate into hematopoietic cells, muscle cells, and ECs [142,143], GBM cells can be directed towards mesenchymal lineage cell types [144]. This might even be more striking with the pluripotent subpopulation of GSCs. Firstly, identical genomic profiles were found in CD133+ tumor cells and their endothelial progeny [145]. Likewise, xenograft studies revealed the sporadic presence of human CD31 expressing cells in the host mouse vasculature. These cells harbored chromosomal aberrations or inherited mutations found in the grafted GSCs, suggesting that a subpart of ECs might arise from GSCs [146]. Of note, tumor-derived endothelial cells (TDEC) that originated from GSCs do not result from cell fusion between ECs and tumor cells [147]. Accordingly, when GSCs were cultured ex vivo under endothelial favorable conditions, they expressed typical endothelial markers, such as CD31, vWF, and Tie-2 [146,147]. These cells were also able to form tubular structures on Matrigel, which is a typical hallmark for endothelial functions [148]. In vitro, hypoxia emerges as an important factor involved in the transdifferentiation process, which nonetheless does not seem to heavily rely on VEGF. Indeed, TDECs are resistant to anti-VEGF therapies, which even increase their frequency [147]. Recently, it has been shown that TDECs differ from tumor-associated vessels and compose molecularly distinct populations [149].

Chemotherapeutic and radiation assaults might further increase GSC subpopulation and emerging TDECs. For instance, irradiated GSCs express Tie2, migrate towards VEGF, and form pseudotubes on Matrigel in vitro [150]. Moreover, TMZ challenge, combined or not with Bevacizumab, potentiates TDEC incorporation in vessels from xenograft models [151,152]. Thus, GSC transdifferentiation could contribute to both resistance to anti-angiogenic therapies and re-vascularization following chemotherapy and/or radiation.

Although how exactly GSC transdifferentiation occurs is not fully elucidated yet, the NOTCH pathway might be involved. The inhibition of NOTCH1 activator b1,4-galactosyltransferase V (b1,4GalTV) prevents GSC transdifferentiation towards ECs [153]. Likewise, NOTCH1 silencing and inhibition block the early stages of GBM cell differentiation into EC intermediates [145]. MicroRNA-34a can also induce transdifferentiation by targeting NOTCH pathway [154].

In addition, GSCs might switch to a pericyte-like program, expressing typical pericyte-enriched markers (α-SMA, NG2, CD248, CD146), without displaying EC features [155]. In addition to sources from the neural crest, mouse studies revealed that a significant amount of brain pericytes derive from neoplastic cells, while GSCs engage towards endothelial lineage via the SDF-1/CXCR4 axis and might in turn differentiate into pericytes, predominantly upon TGF-β challenge [155]. Overall, this might affect the functionality of the vasculature [156,157]. However, pericytes are mostly identified based on expression markers, which are not completely specific and might overlap with other perivascular cell types. How exactly transdifferentiation occurs and persists is still a matter of debate. Because GSCs are highly dynamic and plastic cells, they may co-exist in multiple transition states.

## 8. GBM Resistance to Anti-Angiogenic Therapies

Judah Folkman was among the first to propose the concept of the angiogenic switch, paving the way to the use of anti-angiogenic therapies against cancer [158]. VEGF orchestrates most of the neovascularization processes and many efforts have been developed towards blocking its action. Among the therapeutic arsenal are the monoclonal anti-VEGF-A antibodies Bevacizumab, commercialized under the name Avastin [159,160]. Generally administered in recurrent GBM, this treatment fails however in prolonging patient overall survival. Likewise, the use of anti-angiogenic therapies does not significantly improve survival in newly diagnosed GBM [161]. A combination of anti-angiogenic strategy with chemotherapy might slightly improve the efficacy of chemotherapy treatment alone [162]. Additionally, other inhibitors and antibodies targeting VEGF and VEGFR have been developed, some of which are currently in clinical trials [67].

Multiple potential mechanisms of GBM resistance to anti-angiogenic therapies have been proposed. First, alternative angiogenesis modifiers, such as basic fibroblast growth factor (bFGF), Ang-1/2, and SDF-1 might be used (Table 1). Combination of drugs targeting VEGF and other angiogenic factors seems therefore a relevant therapeutic strategy. Second, tumor cells might also deploy various mechanisms to escape therapeutic insults. For instance, Bevacizumab alters the EV quantity and protein cargo, which further associated with tumor progression and therapeutic resistance [163]. As discussed above, some neovascularization processes, such as vessel co-option and vasculogenesis, are largely independent of VEGF, and therefore insensitive to Bevacizumab. In mouse studies, anti-VEGF therapeutic regimen does not improve animal survival, and appears to promote mesenchymal transition of glioma cells, which further exhibit higher migration, together with elevated infiltration and inflammation in the tumor mass [164].

**Figure 2:**
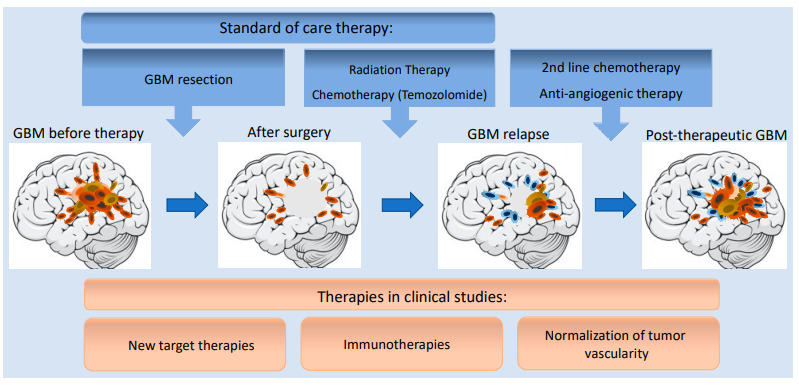
Progression of GBM development during standard-of-care and second line therapies. For GBM patients, standard-of-care therapy usually combines surgery followed radiation and chemotherapy with Temozolomide (TMZ). However, persistant GSCs can acquire more aggressive mesenchymal-like phenotype which might ultimately lead to tumor relapse. In second line treatment, there are few options including chemotherapeutic agents, such as TMZ, and platins, as well as the anti-angiogenic antibodies, Bevacizumab. Novel, promising therapeutic approaches are under development in clinical trials such as new targeted therapies, immunotherapies, and/or vascular normalization.

Importantly, resistance might also loom from the high cell plasticity of GSCs, as exemplified by their transdifferentiation capabilities into ECs and pericytes, a process which, again, does not rely on VEGF signaling. Additionally, GBM standard-of-care (radiation, TMZ) +/− Bevacizumab might foster endothelial transdifferentiation of GSCs [145,150,151,165] (Figure 2). In keeping with this idea, it has been reported that radiation-based pro-angiogenic features of TDECs are supported by the activation of the Tie2 signaling pathway [150,165]. This GSC transdifferentiation process is linked to VM, which has been identified to participate in resistance to anti-angiogenic therapies [166]. Accelerated VM was noticed both in the center and at the periphery of Vatalanib- and Avastin-treated tumors [115,167].

Finally, TDECs also are able to undergo endothelial-to-mesenchymal transition (EndoMT). EndoMT is a recently described process, where ECs lose their endothelial characteristic features and acquire mesenchymal properties. Several factors were shown to initiate EndoMT, including TGF-β, IL-1β, Wnt/β-catenin, NOTCH, as well as hypoxia and oxidative stress [168]. Modified GBM-resident ECs express lower levels of VEGFR and this might ultimately dampen the efficacy of anti-VEGF therapies [169]. However, blocking β-catenin sensitizes to TMZ treatment in mouse GBM model [170,171]. Neo212, a conjugate of TMZ and perillyl alcohol, blocks EndoMT induction in GBM and reverses the mesenchymal phenotype of TDECs by inhibiting both TGF-β and NOTCH pathways. As a consequence, invasiveness and pro-angiogenic properties of brain ECs are reduced [172].

**Figure 3:**
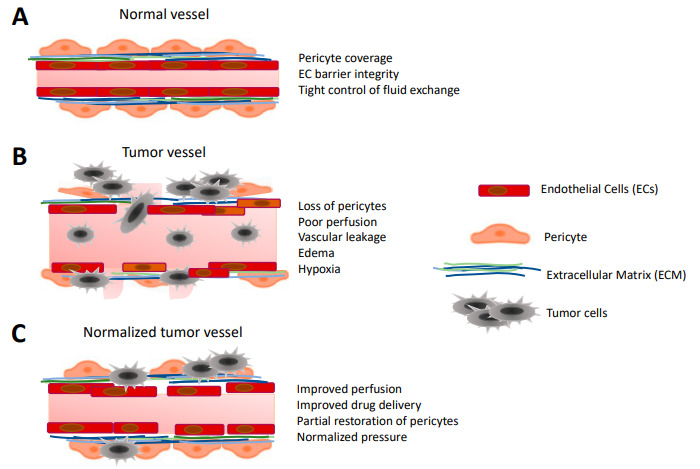
Versatile vessel structures in the course of GBM progression. (**A**) Normal, healthy vessel presents well-organized endothelial cell (EC) layers that are connected with robust junctions and ensheathed with pericytes. (**B**) Abnormal tumor vessel exhibits loss of endothelial junctions leading to increased leakiness and edema. Some of the ECs are transformed, presenting mesenchymal-like phenotype. The pericyte layer is abrogated and the blood brain barrier (BBB) is dysfunctional, hypoxic areas are present. (**C**) Normalized tumor vessel displays better perfusion, partially restored EC and pericyte coverage with semi-functional BBB. Normalized vessels improve drug delivery, while leakiness is decreased.

Additionally, as mentioned above, GBM-associated blood vessels are dilated, tortuous, and leaky with excessively thin basement membrane. Subsequently, tumor vasculature is functionally abnormal with markedly increased interstitial fluid pressure, aggravating hypoxia and acidosis [173]. Under such conditions, radiations are less effective because of the hypoxic environment, while drug delivery is dramatically impeded [20,174,175,176]. This has given birth to the concept of vessel normalization, a strategy aiming to reduce hypoxia, improve drug distribution, and restore immune cell infiltration (Figure 3) [177]. Pre-clinical studies showed that radiations evoke GBM vessel normalization upon transient blocking VEGFR2, and consequently gave better outcomes than radiotherapy alone [178]. Moreover, vascular normalization might be beneficial to restore immune cell homing, in the immunosuppressive GBM environment. For instance, regulatory T-cells were recruited more efficiently upon VEGF and Ang-2 blockade [179]. Other studies showed that targeting endothelial PAK4 promoted GBM vessel normalization, which in turn improved engineered chimeric antigen receptor T cells (CAR-T) infiltration and extended mouse survival [180]. The complexity of GBM vascularization and its heterogeneity thus represent an additional obstacle for anti-angiogenic therapies.

## 9. Conclusions

While hopes have been placed in anti-angiogenic therapies, their promises have been held in check in the case of glioblastoma. In light of the large palette of neovascularization processes at play in GBM, the arsenal of anti-angiogenic therapies needs to be revisited. In keeping with this idea, recent discoveries on alternate mechanisms to generate vessel networks highlighted that vascular normalization might be more efficient than pruning. Combining conventional therapeutic agents (radiation, cytotoxic drugs) and immunotherapies with anti-angiogenic therapies might prove beneficial to improve treatment strategies, patient outcomes, and quality of life.

## Figures and Tables

**Table 1:** List of the key pro- and anti-angiogenic factors involved in GBM vessel formation.

Angiogenic Factors	Role in Neovascularization	Ref
**Vascular Endothelial** **Growth Factor** VEGF-A, -B, C, -D, -E placental growth factor	Key factor of angiogenesis Increases permeability of tumor blood vessels Mediates EC invasion and proliferation Stimulates MMP secretion Acts in synergy with other factors like NRP	[60]
**Platelet-Derived Growth Factor** PDGF-A, -B, -C, -D	Promotes proliferation, migration and tube formation of ECs, pericytes, and smooth muscle cells Contributes to establish a new basement membrane	[61]
**Hepatocyte Growth Factor** HGF	Regulates angiogenesis through MET receptor on ECs Promotes proliferation, migration, survival and ECM	[62]
**Fibroblast Growth Factor** FGF-1, -2	Binds to FGF receptor Interacts with integrin αvβ3 Promotes EC proliferation and ECM degradation Modulates the expression of adhesion molecules Regulated by VEGF, HIF1, bFGF, TGFβ, Ang2	[63]
**Matrix Metalloproteinases** MMP-2,-7,-8, -9	Involved in cell invasion Degrade and remodel ECM	[64]
**Hypoxia-Inducible Factor** 1 HIF-1	Promotes the expression of VEGF, VEGFR, SDF-1, MMPs Assists the recruitment of EPCs, stromal cells, MSCs Triggers angiogenesis, co-option, vasculogenesis and vascular mimicry	[65]
**Angiopoietins** Ang -1,-2	Bind to Tie-2 receptor Ang-1 induces vessel formation and stabilization Ang-2, in the absence of VEGF, is anti-angiogenic and mediates vascular regression and leakiness	[66,67]
**Epithelial Growth Factor** EGF	Binds to EGF receptor, amplified in tumor cells Acts as a pro-angiogenic factor Stimulates VEGF production upon hypoxia Involved in cell proliferation, motility and invasion	[68]
**Tumor Growth Factor β** TGF -β	Involves in EC invasion, differentiation, EndoMT Enhances the expression of pro-angiogenic factors	[69]
**Apelin**	Binds to APJ receptor, expressed in GBM cells and ECs or enriched in tip cells Promotes proliferation and maturation of blood vessels	[11,70]
**Integrins** αvβ3, αvβ5 in GBM cells α1/2/3/5β1, αvβ3 in ECs	Expressed in GBM cells and ECs Support cancer cell adhesion and migration Stabilize EC-tube formation by increasing cell-to-cell adhesion and cell-to-ECM interactions Promote EC proliferation and migration	[71]
**Ephrin ligand A1 and Eph receptor EphA2**	Expressed in GBM cells associated with blood vessels Regulate VEGFR2 expression Promote sprouting angiogenesis Inhibition of EphA2 and VEGFR2 abolished microvessel growth	[72]
